# Social networks and vaccination: a perspective on the essential role of social networks to improve vaccination outcomes in public health

**DOI:** 10.3389/fpubh.2026.1878660

**Published:** 2026-06-16

**Authors:** Nicole Dukers-Muijrers, Rik Crutzen

**Affiliations:** 1 Department of Health Promotion, Care and Public Health Research Institute (CAPHRI), Maastricht University, Maastricht, Netherlands; 2 Department of Health Policy and Development, Living Lab Public Health Mosa (AWPG Mosa), South Limburg Public Health Service, Heerlen, Netherlands

**Keywords:** prevention, public health, social networks, system, vaccination

## Abstract

Vaccines protect against life-threatening diseases and save millions of lives each year in people of all ages, but vaccination rates remain suboptimal and inequitable. Social networks, in which individuals are linked, offer capacity to transmit infectious diseases and protection against them by the diffusion of vaccination behavior. However, social networks are underutilized in public health strategies. This perspective paper advances a network-informed framework that reconceptualizes vaccination behavior as socially embedded. We integrate behavioral drivers with network processes, such as social selection and social influence, and outline how these processes generate network structures that shape vaccination outcomes. We further translate these insights into actionable strategies, including network diagnostics, targeted approaches to alter network structure, and approaches that leverage diffusion. Incorporating social network thinking can strengthen both the effectiveness and equity of vaccination programs.

## Introduction

Vaccines are a major global public health success, protecting people of all ages against more than thirty infectious diseases and preventing an estimated 3.5–5 million deaths annually (1). The goal of vaccination is to reduce morbidity and mortality by limiting disease transmission within populations. However, populations are not homogeneous entities; they are composed of social networks of interconnected individuals. These relationships (or ties) between individuals can serve as pathways for both infectious disease *transmission* and for influence on *behavior* and its antecedents.

Vaccination disrupts *transmission* pathways by reducing susceptibility and infectiousness, altering disease dynamics at the population level. Despite strong infrastructure and vaccine availability in many settings, vaccination uptake remains suboptimal and inequitable, with declining coverage observed globally (2–5). This leaves pockets of susceptible segments of populations where outbreaks can occur, contributing to an avoidable disease burden. Public health responses are often framed in terms of vaccine hesitancy, a motivational state of being conflicted about, or opposed to, getting vaccinated (6). While useful, this framing risks overemphasizing individual decision-making without sufficient attention to the broader social context in which those decisions are embedded (7, 8). This highlights the need to move beyond a false dichotomy between individual and system-level approaches, recognizing individuals as actors embedded within social systems.

Vaccination *behavior* does not occur in isolation. It is shaped through social relationships, shared norms, and access to resources within social networks. Individuals can be embedded in clusters, which are densely connected subsets with shared norms that shape exposure to information, beliefs, and behaviors. The structure of interconnectivity influences how infectious diseases are transmitted and also how information, beliefs, and behaviors diffuse over time. Social network structure influences access to support and information, driving vaccination uptake and inequities. As individuals move through life-course stages and social roles, their network positions and exposures change, further shaping vaccination behaviours. Understanding vaccination as a networked process provides a more complete foundation for intervention.

While prior research has examined vaccine hesitancy, behavioral drivers of vaccination, and diffusion of behaviors through networks, these areas of research have largely developed in parallel. As a result, vaccination is still predominantly understood and addressed at the individual level, with limited integration of how social network structures shape these behaviors, access to resources, and disease dynamics. In this study, we advance a network-informed perspective on vaccination that makes three key contributions. We conceptualize vaccination behavior as a network-embedded process affecting individual decisions, and we link established behavioral drivers to underlying network mechanisms. Then, we translate these insights into a forward-looking public health agenda, outlining how network intervention strategies can be operationalized to improve both uptake and equity. By bridging behavioral science, network theory, and public health practice, this perspective moves toward a more systemic and actionable framework for vaccination strategies.

We draw on a purposive literature search to guide the structure of this perspective distinguishing between (1) network formation mechanisms (such as social selection and homophily) shaping network structure and resulting in clustering, (2) network effects (such as social influence) that describe how beliefs and practices diffuse through these structured social connections, and (3) network-based intervention strategies (such as diagnostics, alteration, and induction) that map and intentionally modify or leverage these network structures and processes.

## Birds of a feather

Social networks are shaped by mechanisms that determine how connections form and how behaviors diffuse through these connections (see Table 1). A key network formation mechanism is social selection, in which people tend to form and maintain relationships (also called ties) with others that offer benefits such as social support and shared identity, while minimizing the costs of maintaining relationships (9, 10). This tendency results in homophily, the observable pattern that connected individuals are alike in terms of sociodemographics, beliefs, and behaviors. Together, these processes lead to clustering: groups of similar individuals within broader networks that are characterized by dense internal connections.

**Table 1:** Changes in the patient’s liver and kidney function test results.

Social network concept	Explanation & relevance to infectious diseases and vaccination
What is a social network from a system perspective?	A social network can be understood as a population, community, or group, composed of individuals (nodes) connected by direct and indirect relationships (ties). These ties form pathways through which infectious diseases are transmitted and beliefs, information, norms, and behaviors diffuse. Network structure and function shape how vaccination behavior evolves at the population level, by enabling or constraining diffusion and access.
What is a social network from an individual perspective?	From an individual perspective, a personal social network consists of the set of social relationships surrounding a person, including close and distant ties across offline and online contexts. These relationships, such as family, friends, and peers, vary in size, diversity and support, and are marked by social selection and social influence processes. Personal networks can provide support and opportunities that enable healthy behavior, such as by offering access to information, norms, resources and support, encouraging self-reliance and participation, and forming a “safety net” to deal with adversity.
What is structure of a social network?	Network structure refers to the pattern of social connections between individuals and their positions within the network. It can be typed by measures such as network size, social isolation, density, diversity, tie-strength, clustering, and centrality and bridging. These structural characteristics determine how individuals are connected and how information, beliefs and behaviors diffuse and how infectious diseases are transmitted.
What is function of a social network?	Network function is described by dynamic processes occurring within the network, including the diffusion of beliefs and behaviors, social influence and the exchange of support and resources. By its function, the social network generates shared resources, such as solidarity, trust, social norms, social cohesion, and collective action, which can shape vaccination decisions.
What are harms and benefits of social connection and social disconnection regarding infectious disease and vaccination.	Social connectedness is the driver for infectious diseases transmission and social connection can amplify supportive and non-supportive beliefs through social influence. At the same time, it can enable access to information, resources, and support that promote vaccination. In contrast, social disconnection may reduce exposure to infectious diseases, but it is also associated with adverse outcomes, including reduced access to care, overall mortality, chronic disease, loneliness, suppressed immune function, and overall poorer health. The weighing of connection and disconnection has important implications for both disease transmission and vaccination behavior.

Vaccination behavior follows these same structural patterns. Social selection and homophily contribute to the emergence of clusters of unvaccinated individuals within otherwise highly vaccinated populations (11). These clusters can form through both strong ties (e.g., family and close friends) and weak ties (e.g., acquaintances). Cluster formation is reinforced by social and structural factors such as age, education, ethnicity, and marital status (12, 13). It is further amplified by geographical proximity, where people with similar backgrounds may interact more frequently (14). Clustering has been observed in measles outbreaks concentrated in geographically and socially clustered unvaccinated populations (15).

Clustering has important consequences for both behavior diffusion and disease transmission. Individuals embedded in unvaccinated clusters may have limited exposure to supportive norms, reduced access to trusted information, and fewer opportunities for indirect protection. At the population level, clustering can sustain rapid transmission within groups even when overall vaccination coverage is high. Clustering may limit transmission between groups. However, connections between clusters, such as bridging ties, could still enable outbreaks to propagate through the broader network (10, 16, 17). Conversely, low clustering may provide some natural protection from outbreaks, as modeled for influenza (18). These dynamics highlight the need to account for network structure, including clustering and connectivity, when designing vaccination strategies.

While social selection explains how structure emerges, social influence explains how beliefs and behaviors diffuse through these network structures. Social influence refers to the mechanisms through which individuals’ beliefs and behaviors are shaped by others, including social norms, modeling, social comparison and cognitive processes through which communicative intentions modulate action-related processing (19, 20). These processes explain why behaviors cluster within networks. Evidence from a range of health behaviors such as smoking, exercise, and diet, demonstrates that norms and practices can diffuse through friends, family members, peers, schools, and workplaces, with effects extending beyond direct ties (21–23). Similar processes likely apply to vaccination, where exposure to supportive or skeptical views can shape attitudes, perceived norms, collective benefits, and decisions (24). Importantly, adopting behaviors such as vaccination often requires reinforcement through multiple exposures. For example, individuals may need to hear the same message multiple times or from different relevant peers, especially when decisions involve risk perceptions, effort, or social consequences (25). The extent of behavior diffusion depends strongly on network structure, including clustering, network density, and the presence of reinforcing or bridging ties, which shape various pathways of social influence (9, 23, 26). In clustered networks, repeated exposure may reinforce existing beliefs, which is important given that a change to vaccination depends on cumulative reinforcement from multiple contacts. Bridging ties can facilitate diffusion of new information to otherwise disconnected clusters.

The relative influence of social selection and social influence varies across contexts, but both processes are key to understanding vaccination patterns and offer a clearer basis for designing effective network-based vaccination strategies in alignment with other relevant drivers of change, for example, by altering exposure to norms and information, and access to resources, reshaping network connections, or leveraging existing ties to support behavior change in combination with other relevant change drivers.

## Change drivers in a network context

To move beyond descriptive accounts of network mechanisms, we connect these to established behavioral frameworks used in vaccination practice. The WHO Behavioral and Social Drivers (BeSD) framework (7) defines four domains that influence vaccination (thinking and feeling, motivation, practical issues, and social processes), each of which operates through network mechanisms: network structure shapes exposure to these drivers, while social influence enables their diffusion.

- Thinking and feeling.
  Includes the cognitive and emotional response to vaccines and the infectious diseases they are targeting, such as perceptions of risk, safety, and effectiveness (10, 27, 28). Such perceptions are influenced by interpersonal communication. Interpersonal communication within networks shapes how individuals interpret disease threats and vaccine benefits through social influence mechanisms. This, in turn, affects decision-making and thus disease transmission.
- Motivation.
  Intentions and willingness to vaccinate are socially reinforced. Individuals exposed to positive attitudes toward vaccination within their networks are more likely to accept vaccination, while exposure to hesitancy can reduce uptake. A review study (29) demonstrated that individuals held more positive vaccination attitudes and were more likely to get vaccinated or to vaccinate their child when they were frequently exposed to positive attitudes and discussed vaccinations with family and friends, while uptake was decreased when family and friends were hesitant to take the vaccine. Studies on COVID-19 further demonstrated that positive vaccination attitudes cluster within personal networks (30), and that discussion networks are polarized by vaccination status, reinforcing hesitancy through social pressure from close associates (31). Another study showed that most couples were concordant in vaccination status, yet a substantial minority remain discordant mainly due to safety concerns (32).
- Practical issues, information, and other types of social support.
  Access to vaccination is influenced by both structural factors and social support. Networks can facilitate access through shared information, logistical assistance (e.g., transport or costs), and encouragement and emotional support, while also shaping trust in healthcare systems and services (33, 34). Larger and more diverse networks with both strong ties (family and friends) and weak ties (other types of contacts, such as those offering new information) have been associated with higher vaccination intention and uptake during COVID-19 (35, 36). Positive individual vaccination experiences, shaped within social networks and shared among ties, can reduce fear, anxiety, and stress, while fostering empowerment, emotional safety, and sustained trust in vaccination services within the community.
- Social processes.
  Perceived social norms, both descriptive norms (what most people do) and injunctive norms (what most people approve or disapprove of), play a central role in shaping vaccination and other behaviors (37). Social norms are a key mechanism of social influence. Individuals are influenced by these norms as well as by a desire for social acceptance and responsibility toward others. Social influence is driven by a desire for accurate understanding, social acceptance, and maintaining a positive self-concept (38–40). In a US study, the belief in having social responsibility to get vaccinated for COVID-19 to protect others had the largest effect on vaccination intention and mediated the effects of perceived risk and demographics (41). Interventions that leverage social norms and collective responsibility, next to individual risk perception, can therefore be particularly effective.

These domains of drivers are not by default integrated into explicit network-based approaches in research, modeling, or intervention design, while we argue that it would be beneficial to do so.

## Social network interventions

Network processes provide concrete entry points for intervention efforts. Including social networks in public health interventions can improve effectiveness and reach of these interventions, across a range of health outcomes (24), by identifying key network structures (diagnostics), modifying connections (alteration), and leveraging existing ties to diffuse behaviors (induction) as described by Valente (42).

- Network diagnostics or mapping (identifying and targeting individuals or groups).
  Network diagnostics focus on identifying network structure, including clustering, centrality, and bridging positions. Network mapping can identify clusters of low vaccination uptake, highly connected individuals, and groups with increased transmission relevance. These may include marginalized populations, highly connected community members, healthcare workers, or other key occupational groups, such as in COVID-19, influenza or MPOX vaccination (43–49). Targeting key individuals or clusters of densely connected individuals can improve intervention efficiency compared to untargeted approaches. Understanding network connections can inform targeted alteration and induction strategies, for example, to promote behavioral change within an unvaccinated cluster through multiple exposures. Simulation studies demonstrated that clustered seeding of vaccine supporters in central positions (e.g., trusted persons having many connections) or in bridging positions could increase vaccine uptake more effectively than random seeding or group-based targeting alone (23, 49), while empirical studies in other health domains demonstrate effectiveness of both key individual and group-based approaches (50). Mapping improves when overlapping networks (e.g., offline or online, or work, school, medical, and private settings) are considered (49). Alternative strategies, such as targeting nominated friends of selected individuals has also been shown to enhance intervention diffusion (51) without requiring full network mapping. Vaccinating individuals in central network positions, such as highly connected persons, can reduce transmission more effectively than random vaccination, supporting herd immunity (52). Individuals in central positions can amplify intervention effects to promote vaccination. These include parents, opinion leaders, caregivers, or intermediaries who are trusted sources of information in the community. Brokers (which can be strong or weak ties) facilitate diffusion across otherwise disconnected groups (10). In addition, the identification of people on the periphery of the community, or those who are more disconnected, may also be relevant since they are potentially excluded from services or the positive supports derived from community participation (42).
- Network alteration (changing network structure).
  Network alteration aims to modify network structures or strengthen connections between communities and health systems. Examples of adding connections include school-based vaccination or outreach programs, online peers, and community hubs that embed services within social contexts and improve access (53). Modeling studies suggest that introducing positive vaccine nodes or disrupting pathways of influence can prevent the formation of anti-vaccine clusters (54, 55). Broader public health measures, such as physical distancing, further illustrate how modifying contacts can affect disease transmission (56, 57). Although vaccination alters transmission dynamics within networks, deliberate network alteration remains relatively underexplored in vaccination behavior research. An alternative example of network alteration relevant to vaccination campaigns may be targeting those individuals who are embedded across multiple networks (e.g., family, work, and online), but who are not necessarily the most central in any single layer, to alter how information, access, and influence diffuse through the network, without necessarily changing formal network ties (49).
- Network induction (leveraging existing ties to diffuse behavior).
  Network induction leverages existing ties to stimulate the diffusion of vaccination information, beliefs, and behaviors through existing social ties, thereby extending impact beyond directly targeted individuals. For example, individuals may be encouraged to share vaccination information with peers, an approach often used to reach hidden or underserved populations, through respondent-driven (peer-referral), geospatial, and venue-based sampling, although evidence on the application and impact of these adaptive sampling approaches remains limited (58). A review of peer-interventions, primarily in students, for HPV, COVID-19 and influenza vaccines, demonstrated positive effects on vaccine uptake or beliefs, especially when combined with involvement from healthcare professionals (59). Other approaches, including geospatial or venue-based strategies and dialogue-based interventions with reminders or incentives, may further improve uptake (60). These approaches involve interactive communication to address concerns, build trust, and support informed decision-making, for example through community meetings, peer discussions, or clinical encounters in trusted settings. Such mechanisms can be particularly important given evidence that vaccine-related misinformation is often socially reinforced and resistant to simple factual correction (61). Social norms are a key mechanism underlying induction effects. Individuals are influenced by (their perceptions of) behaviors within their networks, and reinforcement from multiple relevant peers or diverse social contexts is linked to vaccination. Vaccinated individuals perceive stronger vaccination norms and reinforcing behavior, while uptake is often underestimated among peers (62), correcting such misperceptions may increase vaccination intentions (63). In addition, having more frequent discussions about vaccination was linked to higher uptake (29).

Effective vaccination strategies therefore require community-specific outreach that addresses underlying inequities and combine multiple intervention types aligned with network-strategies and key vaccine drivers such as confidence, trust, and social norms. Despite promising evidence, network-based approaches remain underused in practice, highlighting the need for a more network-informed vaccination agenda.

## Network-informed vaccination agenda

Current public health approaches have limited attention to how social connections shape vaccination behavior and access. To strengthen uptake and equity, we propose the following network-informed agenda, which links change drivers, network processes, network structure and vaccination outcomes, and which can be operationalized from local community outreach initiatives to national research and surveillance levels.

**Figure 1:**
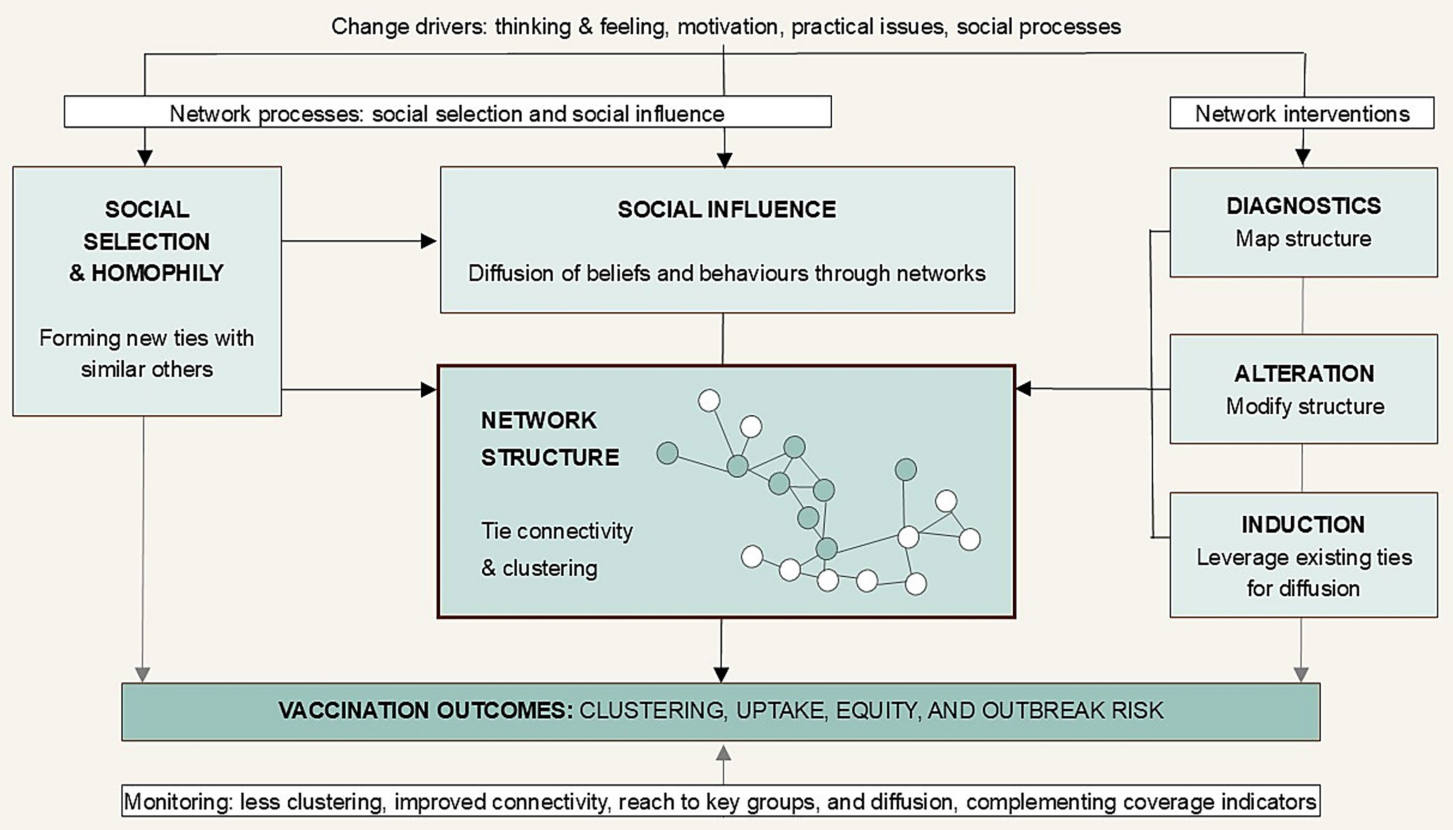
**Vaccination as a networked process.** Behavioral drivers of vaccination (thinking and feeling, motivation, practical issues, and social processes; based on the WHO Behavioral and Social Drivers framework) operate through social networks via two key processes: social selection, which structures connections between similar individuals, and social influence, through which beliefs and behaviors diffuse across ties. Together, these processes, reinforced by homophily, give rise to network structure characterized by connectivity and clustering, which shape vaccination outcomes. Network-informed intervention strategies act on these structures through diagnostics (identifying clusters and key actors), alteration (modifying connections and access), and induction (leveraging existing ties for diffusion). Integrating social network perspectives can improve uptake and equity while reducing clustering and outbreak risk.

1. Reframe vaccination as a network process.
  Vaccination behavior is shaped through social relationships, norms, and shared resources. Moving beyond a focus on individual hesitancy, interventions should address how beliefs, motivation, access, and social influence operate within social networks. For example, vaccination decisions among parents are influenced by discussions in parent groups, schools, or online communities, where repeated interaction with similar others reinforce shared norms, contributing to network structure patterns such as clustering. This perspective supports strategies that explicitly leverage social influence, social responsibility, and indirect protection.
2. Apply social network diagnostics.
  Mapping vaccination uptake, beliefs, and interactions within social networks can provide valuable insights. It can identify unvaccinated clusters, underserved groups, high-risk settings (e.g., geographic areas, workplaces, and care facilities), and influential individuals. For instance, measles outbreaks have been linked to geographically and socially clustered communities with low uptake. Data collection methods may include surveys, personal network assessment, contact mapping, or adaptive sampling, using measures such as degree, density, bridging, and clustering, to enable more precise and context-specific design of interventions.
3. Target clusters.
  Interventions should focus on densely connected groups with lower vaccination uptake, where shared beliefs and limited exposure to alternative perspectives may reinforce non-vaccination. Reaching interconnected individuals simultaneously and repeatedly increases the likelihood of shifting norms and behaviors. For example, targeted outreach in specific community or neighborhood groups rather than mass campaigns to disrupt clustered patterns of non-vaccination. Integrating network insights with demographic and geographic data can improve outreach to underserved clusters and more equitable resource allocation.
4. Leverage key social actors for diffusion and social norms.
  Individuals or institutions in key network positions, such as community leaders, healthcare workers, peer leaders, and well-connected community members, can accelerate the diffusion of vaccination behaviors and extend influence across social boundaries. For example, engaging trusted healthcare workers or opinion leaders as messengers or training peer ambassadors in work or school settings to increase behavior diffusion. Peer-led approaches, particularly when supported by trusted institutions or individuals, can foster dialogue, strengthen norms, and enhance acceptance.
5. Employ network alteration methods.
  Vaccination programs can modify network structure and strengthen connections between communities and health systems by embedding services within existing social structures. Examples include mobile clinics in neighborhoods or in community centers and outreach initiatives. Such network-based approaches can reduce structural barriers, such as transport, financial, and social support (including information) gaps, to increase access to diverse and trusted information and resources for structurally isolated or underserved network positions and help prevent formation or persistence of unvaccinated clusters.
6. Institutionalize network-informed vaccination research and practice.
  Sustained impact requires integrating network perspectives into research, policy, and implementation. This includes incorporating social network data into surveillance, combining social and epidemiological modeling, and evaluating interventions using broader metrics. For example, monitoring whether interventions reduce clustering on non-vaccination, increase connections, improve reach into key groups and increase diffusion of trust and supportive vaccination beliefs, complement standard coverage indicators. Strengthening interdisciplinary collaboration and embedding these approaches in vaccination programs will support effective and equitable strategies (see Figure 1).

## Discussion

This paper provides an integrated framework linking network structure, behavioral drivers, and intervention design. We show that vaccination outcomes are not solely the result of individual decisions but also emerge from the structure and dynamics of social networks. Processes such as social selection, social influence, clustering of non-vaccination, and unequal access to information, support, and resources create persistent coverage gaps. These mechanisms can sustain the risk of localized outbreaks, even in populations with high overall vaccination rates.

Despite this, most public health strategies pay limited attention to how people are socially connected and how behaviors diffuse through these connections. This leaves clear opportunities untapped. For example, interventions could be more effective if they explicitly targeted interconnected groups, shifted norms within clusters, or leveraged individuals or organizations in key network positions. Network-based approaches address these gaps, by aligning vaccination strategies with real-world patterns of social interaction rather than relying solely on aggregate individual data. This alignment can increase both efficiency and equity, particularly for underserved groups who are missed by conventional approaches.

At the same time, translating network-based approaches into practice raises several important challenges.

First, implementing network-based strategies at population scale can be resource-intensive and operationally complex. Mapping social networks, identifying clusters, and targeting key structures or actors may require substantial data collection, analytic capacity, and integration within existing public health surveillance systems. These resources are not always available, and mapping networks in large or fragmented populations can be difficult when data infrastructures are limited.

Second, the use of network data raises ethical and privacy considerations. Information on social ties, behaviors and beliefs can be sensitive data. Inadequate data protection may undermine trust in public health systems. Ensuring informed consent, data minimization, and secure data governance is therefore essential.

Third, targeting unvaccinated clusters carries a risk of stigma and unintended social consequences. Labeling specific communities, schools, or social groups may unintentionally reinforce marginalization or reduce trust. This is particularly relevant for already underserved or vulnerable populations, including culturally, religiously, or sexually defined groups. Fully engaging communities in the design and implementation of approaches to vaccination is therefore critical to ensure culturally sensitive and acceptable approaches (64).

Fourth, analysis of social networks may introduce bias. For example, online collected network data do not fully represent in-person interactions and may under-represent certain demographic groups, such as individuals with limited digital health literacy. Monitoring survey-based network data can likewise introduce selection bias and may exclude less socially connected individuals. Careful consideration of representativeness and data limitations is therefore needed.

Fifth, social networks are dynamic, requiring additional efforts to capture and address them accurately over time. Changes in everyday life structure and daily hassles shape people’s network as well as their behavior. Roles such as partnership, caregiving, and having paid work shape a variety of lifestyle behaviors, including alcohol consumption (65) and likely also vaccination. Incorporating these changing networked life-course roles is a future research direction that supports a vaccination approach that spans age and generations (66).

Sixth, the applicability of network informed approaches may vary across settings. In high-resources settings, access to data, analytic tools, and established infrastructures may facilitate implementation. In contrast, low-resource settings may require alternative approaches, such as qualitative network assessment or leveraging existing community health structures. Adapting strategies to local contexts is therefore essential.

Finally, while network-based interventions show high promise, empirical evidence on their effectiveness remains uneven. There is a need for evaluation across different infectious diseases, populations, and settings, to determine which strategies are most effective, acceptable, and scalable.

Taken together, these challenges highlight the importance of careful, context-sensitive implementation and ongoing evaluation.

Adopting a network perspective strengthens individual-focused frameworks by situating behaviors within their social context. This offers a more complete basis for understanding and influencing vaccination uptake. In the context of globally declining vaccination rates and persistent inequities, integrating social network thinking into research, policy, and practice, through targeted data collection, interdisciplinary collaboration, and embedding these approaches within public health systems, is an important step forward to strengthening population-level protection against vaccine-preventable infectious diseases.

## Data Availability

The original contributions presented in the study are included in the article/supplementary material, further inquiries can be directed to the corresponding author.

## References

[1] World Health Organization. Vaccines and Immunization. Geneva: World Health Organization. Available online at: https://www.who.int/health-topics/vaccines-and-immunization (Accessed May 2026).

[2] World Health Organization. Immunization Coverage. Geneva: World Health Organization (2021). Available online at: https://www.who.int/news-room/fact-sheets/detail/immunization-coverage (Accessed May 2026).

[3] Chandir S Siddiqi DA Mehmood M Setayesh H Siddique M Mirza A . Impact of COVID-19 pandemic response on uptake of routine immunizations in Sindh, Pakistan: an analysis of provincial electronic immunization registry data. Vaccine. (2020) 38:7146–55. doi: 10.1016/j.vaccine.2020.08.019, 32943265 PMC7428732

[4] McDonald HI Tessier E White JM Woodruff M Knowles C Bates C . Early impact of the coronavirus disease (COVID-19) pandemic and physical distancing measures on routine childhood vaccinations in England, 2020. Euro Surveill. (2020) 25:2000848. doi: 10.2807/1560-7917.ES.2020.25.19.2000848, 32431288 PMC7238742

[5] Shapiro GK Gottfredson N Leask J Wiley K Ganter-Restrepo FE Jones SP . COVID-19 and missed or delayed vaccination in 26 middle- and high-income countries: an observational survey. Vaccine. (2022) 40:945–52. doi: 10.1016/j.vaccine.2021.12.016, 35039193 PMC8687753

[6] Bussink-Voorend D Hautvast JLA Vandeberg L Visser O Hulscher MEJL. A systematic literature review to clarify the concept of vaccine hesitancy. Nat Hum Behav. (2022) 6:1634–48. doi: 10.1038/s41562-022-01431-6, 35995837

[7] World Health Organization. Understanding the behavioural and social drivers of vaccine uptake: WHO position paper-May 2022. Wkly Epidemiol Rec. (2022) 97:209–24. doi: 10.3760/cma.j.cn112150-20220706-00686

[8] Chater N Loewenstein G. The i-frame and the s-frame: how focusing on individual-level solutions has led behavioral public policy astray. Behav Brain Sci. (2023) 46:e147. doi: 10.1017/S0140525X22002023, 36059098

[9] Jackson MO. Social and Economic Networks. Princeton, NJ: Princeton University Press (2008).

[10] Nunner H Buskens V Teslya A Kretzschmar M. Health behavior homophily can mitigate the spread of infectious diseases in small-world networks. Soc Sci Med. (2022) 312:115350. doi: 10.1016/j.socscimed.2022.115350, 36183539

[11] Stefkovics Á Albert F Ligeti AS Dávid B Rudas S Koltai J. Vaccination homophily in ego contact networks during the COVID-19 pandemic. Sci Rep. (2024) 14:15515. doi: 10.1038/s41598-024-65986-2, 38969667 PMC11226437

[12] Bish A Michie S. Demographic and attitudinal determinants of protective behaviours during a pandemic: a review. Br J Health Psychol. (2010) 15:797–824. doi: 10.1348/135910710X485826, 20109274 PMC7185452

[13] McPherson M Smith-Lovin L Cook JM. Birds of a feather: homophily in social networks. Annu Rev Sociol. (2001) 27:415–44. doi: 10.1146/annurev.soc.27.1.415

[14] Alvarez-Zuzek LG Zipfel CM Bansal S. Spatial clustering in vaccination hesitancy: the role of social influence and social selection. PLoS Comput Biol. (2022) 18:e1010437. doi: 10.1371/journal.pcbi.1010437, 36227809 PMC9562150

[15] Brownwright TK Dodson ZM van Panhuis WG. Spatial clustering of measles vaccination coverage among children in sub-Saharan Africa. BMC Public Health. (2017) 17:957. doi: 10.1186/s12889-017-4961-9, 29246217 PMC5732449

[16] Are E Card K Colijn C. The role of vaccine status homophily in the COVID-19 pandemic: a cross-sectional survey with modelling. BMC Public Health. (2024) 24:472. doi: 10.1186/s12889-024-17957-5, 38355444 PMC10868109

[17] European Centre for Disease Prevention and Control. Increase of Pertussis Cases in the EU/EEA. Stockholm: ECDC (2024).

[18] Edge R Heath J Rowlingson B Keegan TJ Isba R. Seasonal influenza vaccination amongst medical students: a social network analysis based on a cross-sectional study. PLoS One. (2015) 10:e0140085. doi: 10.1371/journal.pone.0140085, 26452223 PMC4599893

[19] de la Haye K Green HD Jr Kennedy DP Pollard MS Tucker JS. Selection and influence mechanisms associated with marijuana initiation and use in adolescent friendship networks. J Res Adolesc. (2013) 23:474–86. doi: 10.1111/jora.12018, 24187477 PMC3811150

[20] Kashima Y Bekkering H Kashima ES. Communicative intentions can modulate the linguistic perception-action link. Behav Brain Sci. (2013) 36:361–2. doi: 10.1017/S0140525X12002610, 23790071

[21] Christakis NA Fowler JH. Social contagion theory: examining dynamic social networks and human behavior. Stat Med. (2013) 32:556–77. doi: 10.1002/sim.5408, 22711416 PMC3830455

[22] Montgomery SC Donnelly M Bhatnagar P Carlin A Kee F Hunter RF. Peer social network processes and adolescent health behaviors: a systematic review. Prev Med. (2020) 130:105900. doi: 10.1016/j.ypmed.2019.105900, 31733224

[23] Zhang J Centola D. Social networks and health: new developments in diffusion, online and offline. Annu Rev Sociol. (2019) 45:91–109. doi: 10.1146/annurev-soc-073117-041421

[24] Hunter RF de la Haye K Murray JM Badham J Valente TW Clarke M . Social network interventions for health behaviours and outcomes: a systematic review and meta-analysis. PLoS Med. (2019) 16:e1002890. doi: 10.1371/journal.pmed.1002890, 31479454 PMC6719831

[25] Centola D Macy M. Complex contagions and the weakness of long ties. Am J Sociol. (2007) 113:702–34. doi: 10.1086/521848

[26] Andres E Ódor G Iacopini I Karsai M. Distinguishing mechanisms of social contagion from local network view. Npj Complex Syst. (2025) 2:8. doi: 10.1038/s44260-025-00034-2, 40051436 PMC11879858

[27] Lau JTF Yang X Tsui HY Kim JH. Impacts of SARS on health-seeking behaviors in the general population in Hong Kong. Prev Med. (2005) 41:454–62. doi: 10.1016/j.ypmed.2004.11.023, 15917041 PMC7119319

[28] Teslya A Nunner H Buskens V Kretzschmar ME. The effect of competition between health opinions on epidemic dynamics. PNAS Nexus. (2022) 1:pgac260. doi: 10.1093/pnasnexus/pgac260, 36712334 PMC9802282

[29] Konstantinou P Georgiou K Kumar N Kyprianidou M Nicolaides C Karekla M . Transmission of vaccination attitudes and uptake based on social contagion theory: a scoping review. Vaccine. (2021) 9:607. doi: 10.3390/vaccines9060607, 34198885 PMC8229666

[30] Hâncean MG Lerner J Perc M Molina JL Geantă M. Assortative mixing of opinions about COVID-19 vaccination in personal networks. Sci Rep. (2024) 14:3385. doi: 10.1038/s41598-024-53825-3, 38336858 PMC10858210

[31] Amlani S Kiesel S Butters R. Polarization in COVID-19 vaccine discussion networks. Am Polit Res. (2023) 51:260–73. doi: 10.1177/1532673X221148670, 38603344 PMC9813645

[32] Schmaling KB. Couples and COVID-19 vaccination: frequency and reasons for discordance. Vaccine. (2022) 40:1913–7. doi: 10.1016/j.vaccine.2022.02.055, 35216841 PMC8853825

[33] Larson HJ Clarke RM Jarrett C Eckersberger E Levine Z Schulz WS . Measuring trust in vaccination: a systematic review. Hum Vaccin Immunother. (2018) 14:1599–609. doi: 10.1080/21645515.2018.1459252, 29617183 PMC6067893

[34] Wells K Moore KL Bednarczyk R. Supporting immunization programs to address COVID-19 vaccine hesitancy. Vaccine. (2022) 40:2819–22. doi: 10.1016/j.vaccine.2022.03.039, 35397947 PMC8958158

[35] Steijvers LCJ van Bilsen CJA Wagner S Stutterheim SE Crutzen R Ruiter RAC . Social networks and COVID-19 vaccination intention in Dutch middle-aged and older adults: the SaNAE study. Vaccine X. (2024) 20:100562. doi: 10.1016/j.jvacx.2024.100562, 39399819 PMC11466667

[36] van Bilsen CJA Steijvers LCJ Wagner S Pagen DME Wijnen SMCE Konings K . Social networks, environmental, and individual factors associated with COVID-19 vaccination and booster uptake: the PRIME cohort study. Vaccine X. (2025) 23:100626. doi: 10.1016/j.jvacx.2025.100626, 38826717

[37] Dempsey RC Wood AM. Perceived social norms and vaccine hesitancy. Curr Dir Psychol Sci. (2025) 34:357–64. doi: 10.1177/09637214251340023

[38] Chen X Li S Zhang Y Zhai Y Zhang Z Feng C. Different drives of herding: motivations underlying social conformity. Psych J. (2022) 11:247–58. doi: 10.1002/pchj.515, 35080146

[39] Cialdini RB Goldstein NJ. Social influence: compliance and conformity. Annu Rev Psychol. (2004) 55:591–621. doi: 10.1146/annurev.psych.55.090902.142015, 14744228

[40] Swann WB Jetten J Gómez A Whitehouse H Bastian B. When group membership gets personal: a theory of identity fusion. Psychol Rev. (2012) 119:441–56. doi: 10.1037/a0028589, 22642548

[41] Fung H Sgaier SK Huang VS. Discovery of interconnected causal drivers of COVID-19 vaccination intentions in the US. Sci Rep. (2023) 13:6988. doi: 10.1038/s41598-023-33745-4, 37193707 PMC10188432

[42] Valente TW. Network interventions. Science. (2012) 337:49–53. doi: 10.1126/science.1217330, 22767921

[43] Memedovich A Orr T Hollis A Salmon C Hu J Zinszer K . Social network risk factors and COVID-19 vaccination: a cross-sectional survey study. Vaccine. (2024) 42:891–911. doi: 10.1016/j.vaccine.2024.01.012, 38238114

[44] Saunders HA Schwartz JM. COVID-19 vaccination strategies depend on the underlying network of social interactions. Sci Rep. (2021) 11:24051. doi: 10.1038/s41598-021-03167-1, 34912001 PMC8674282

[45] Schumacher S Salmanton-García J Liekweg A Rolfes M Seidel D Mellinghoff SC . Increasing influenza vaccination coverage in healthcare workers. Infection. (2023) 51:1417–29. doi: 10.1007/s15010-023-02007-w, 36853494 PMC9972307

[46] Ayers CK Kondo KK Williams BE Kansagara D Advani SM Smith M . Disparities in H1N1 vaccination rates: a systematic review. J Gen Intern Med. (2021) 36:1734–45. doi: 10.1007/s11606-021-06715-7, 33791935 PMC8011776

[47] Alavian N Mourad A Woodhouse EW Niehaus E Cunningham H Zavala S . Disparities in Mpox vaccination among priority populations during the 2022 outbreak. Open Forum Infect Dis. (2023) 10:ofad434. doi: 10.1093/ofid/ofad434, 37662451 PMC10472485

[48] Dukers-Muijrers NHTM Evers Y Widdershoven V Davidovich U Adam PCG op de Coul ELM . Mpox vaccination willingness, determinants, and communication needs in gay, bisexual, and other men who have sex with men, in the context of limited vaccine availability in the Netherlands (Dutch Mpox-survey). Front Public Health. (2023) 10:1058807. doi: 10.3389/fpubh.2022.1058807, 36684959 PMC9850232

[49] Fügenschuh M Fu F. Overcoming vaccine hesitancy by multiplex social network targeting. Appl Netw Sci. (2023) 8:67. doi: 10.1007/s41109-023-00595-y, 37745797 PMC10514145

[50] Hunter R de la Haye K Badham J Valente T Clarke M Kee F. Social network interventions for health behaviour change. Lancet. (2017) 390:S47. doi: 10.1016/S0140-6736(17)32982-3, 31479454 PMC6719831

[51] Kim DA Hwong AR Stafford D Hughes DA O'Malley AJ Fowler JH . Social network targeting to maximise population behaviour change: a cluster randomised controlled trial. Lancet. (2015) 386:145–53. doi: 10.1016/S0140-6736(15)60095-2, 25952354 PMC4638320

[52] Pastor-Satorras R Vespignani A. Immunization of complex networks. Phys Rev E. (2002) 65:036104. doi: 10.1103/PhysRevE.65.036104, 11909162

[53] Pivette M Taha MK Barret AS Polard E Hautier MB Dufour JB . Targeted vaccination campaigns of teenagers after clusters of invasive meningococcal disease, France, 2017. BMC Public Health. (2020) 20:1382. doi: 10.1186/s12889-020-09487-7, 32912190 PMC7488129

[54] Alahmadi S Hoyle R Head M Brede M. Modelling mitigation of anti-vaccine opinion propagation. PLoS One. (2025) 20:e0318544. doi: 10.1371/journal.pone.0318544, 40111968 PMC11925286

[55] Alahmadi S Hoyle RB Head M Brede M. Optimal intervention to combat anti-vaccine social contagion. Appl Netw Sci. (2025) 10:59. doi: 10.1007/s41109-025-00718-7

[56] Nishi A Dewey G Mengual M Ando H Cassol-Pawson N Endo A. Infectious disease control as network interventions. Discov Soc Sci Health. (2025) 5:72. doi: 10.1007/s44155-025-00217-1

[57] Robins G Lusher D Broccatelli C Bright D Gallagher C Karkavandi MA . Multilevel network interventions: goals, actions, and outcomes. Soc Networks. (2023) 72:108–20. doi: 10.1016/j.socnet.2022.09.005, 36188126 PMC9504355

[58] Koyuncu A Ishizumi A Daniels D Jalloh MF Wallace AS Prybylski D. Adaptive sampling to reach disadvantaged populations for immunization. Vaccine. (2023) 11:424. doi: 10.3390/vaccines11020424, 36851301 PMC9961530

[59] Gobbo ELS Hanson C Abunnaja KSS van Wees SH. Peer-based education interventions and vaccination acceptance: a systematic review. BMC Public Health. (2023) 23:1354. doi: 10.1186/s12889-023-16294-3, 37452295 PMC10349425

[60] Liu J Zhang Y Zhang H Tan H. Estimating the effects of interventions on increasing vaccination uptake. BMJ Glob Health. (2025) 10:e017142. doi: 10.1136/bmjgh-2024-017142, 40204467 PMC11987150

[61] Ecker UKH Lewandowsky S Cook J Schmid P Fazio LK Brashier N . The psychological drivers of misinformation belief and its resistance to correction. Nat Rev Psychol. (2022) 1:13–29. doi: 10.1038/s44159-021-00006-y

[62] Yewell D Bentley R Horne B. Perceived social influence on vaccination decisions: a COVID-19 case study. SN Soc Sci. (2024) 4:125. doi: 10.1007/s43545-024-00929-2

[63] Moehring A Collis A Garimella K Rahimian MA Aral S Eckles D. Providing normative information increases intentions to accept a COVID-19 vaccine. Nat Commun. (2023) 14:126. doi: 10.1038/s41467-022-35052-4, 36624092 PMC9828376

[64] World Health Organization Immunization Agenda 2030: A Global Strategy to Leave No-One Behind. Available online at: https://www.who.int/publications/m/item/immunization-agenda-2030-a-global-strategy-to-leave-no-one-behind (Accessed May 2026).

[65] Crutzen R Knibbe RA. A Dutch panel study on the relation between structure of everyday life, daily hassles, and alcohol consumption. BMC Public Health. (2012) 12:1068. doi: 10.1186/1471-2458-12-1068, 23231767 PMC3533985

[66] Balsells E Ghiselli M Hommes C Nascimento Lins de Oliveira B Rosado-Valenzuela AL Vega E. Rethinking immunization programs through the life course approach. Front Public Health. (2024) 12:1355384. doi: 10.3389/fpubh.2024.1355384, 38487192 PMC10937433

